# Prediction of cardiopulmonary events using the STOP-Bang questionnaire in patients undergoing bronchoscopy with moderate sedation

**DOI:** 10.1038/s41598-020-71314-1

**Published:** 2020-09-02

**Authors:** Jaeyoung Cho, Sun Mi Choi, Young Sik Park, Chang-Hoon Lee, Sang-Min Lee, Chul-Gyu Yoo, Young Whan Kim, Jinwoo Lee

**Affiliations:** 1 Division of Pulmonary and Critical Care Medicine, Department of Internal Medicine, Seoul National University Hospital, Seoul, Republic of Korea; 2 Department of Internal Medicine, Seoul National University College of Medicine, 103 Daehak-ro, Seoul, 03080 Republic of Korea

**Keywords:** Sleep disorders, Epidemiology

## Abstract

The objective was to evaluate the prevalence of patients at a high risk of having OSA by using a screening questionnaire and to investigate whether the questionnaire can predict patients who are at risk of cardiopulmonary events occurring during a bronchoscopy under sedation. We prospectively enrolled consecutive adult patients who underwent flexible bronchoscopies under moderate sedation. The snoring, tiredness, observed apnea, high blood pressure-body mass index, age, neck circumference and gender (STOP-Bang) questionnaire was used to identify patients at a high (score ≥ 3 of 8) or low risk (score < 3 of 8) of having OSA. The cardiopulmonary events included hypoxemia and hypotension. Multivariable logistic regression was performed with variables selected by the least absolute shrinkage and selection operator. The prevalence of a STOP-Bang score of ≥ 3 was 67.2% (195/290), and 36.9% (107/290) experienced cardiopulmonary events. The multivariable analysis adjusting for chronic obstructive pulmonary disease, chronic kidney disease, baseline SpO_2_, and procedure time revealed that a STOP-Bang score of ≥ 3 was significantly associated with cardiopulmonary events in a subgroup of patients without a history of cerebrovascular disease (adjusted odds ratio, 1.94; 95% confidence interval, 1.06–3.54). The STOP-Bang questionnaire can predict cardiopulmonary events occurring during this procedure.

**Trial registration**: NCT03325153.

## Introduction

Obstructive sleep apnea (OSA) is characterized by repetitive episodes of complete or partial upper airway obstruction occurring during sleep^1^. It is suggested that about 1 billion adults worldwide may be affected by OSA^2^. The prevalence of OSA—defined as more than five apneas or hypopneas per hour of sleep plus excessive daytime sleepiness—was 12.5% in men and 5.9% in women in the middle to old age general population^3,4^.

Overnight polysomnography is the gold standard for the diagnosis of OSA. However, it is time-consuming, labor-intensive, and costly^5,6^. Although patients with OSA are at an increased risk of developing perioperative cardiac and pulmonary complications^7–10^, approximately 60% of those with moderate to severe OSA are not recognized preoperatively^11,12^.

Patients with OSA may experience obstructive episodes during procedures while under moderate sedation^13^. However, the rate of adverse events in undiagnosed OSA patients undergoing bronchoscopies under moderate sedation remains poorly described. In early 2000, Sharma et al*.* reported that OSA was detected in three-quarters of 23 patients without a previous diagnosis of sleep apnea while undergoing outpatient bronchoscopy or colonoscopy^14^. Recently, a study with obese patients having bronchoscopy under moderate sedation showed that the incidences of procedural complications were similar between obese and non-obese patients. However, a subset (7%) of patients with previous polysomnography-proven OSA were more likely to have earlier termination of bronchoscopy than patients without diagnosed OSA^15^. All procedures terminated early in patients with OSA were due to hypoxemia.

Here, we aimed to evaluate the prevalence of patients at a high risk of having OSA by using a screening questionnaire and to investigate whether the questionnaire can predict patients who are at risk of experiencing cardiopulmonary events during a bronchoscopy under moderate sedation.

## Material and methods

### Study design and patients

We prospectively enrolled consecutive adult patients undergoing flexible bronchoscopies under moderate sedation between 27 December 2016 and 11 December 2017 at the Bronchoscopy Center at Seoul National University Hospital, a 1,780-bed tertiary teaching hospital and the one of the largest referral centers in South Korea. We excluded patients with a previous diagnosis of OSA, an American Society of Anesthesiologists (ASA) physical status of > 3, a tracheostomy tube, a baseline pulse oximeter oxygen saturation (SpO_2_) of < 90%, the need for supplemental oxygen before the procedure, or an inability to provide informed consent. We also excluded patients undergoing bronchoalveolar lavage (BAL), radial and convex probe endobronchial ultrasound (EBUS), navigational bronchoscopy, and interventional bronchoscopy (balloon dilatation, endobronchial debulking of tumors with or without electrocautery/thermal laser, cryotherapy or cryoextraction, airway stent placement or manipulation, photodynamic therapy, and brachytherapy). BAL is distinguished from bronchial washing, in that a bronchoscope is wedged into a position where the lumen of the small bronchus is occluded by the bronchoscope to minimize sampling from the large airways^16^. Initially, we excluded outpatients undergoing bronchoscopy due to the shortage of research personnel to collect various information about outpatients within the short duration of the outpatients’ hospital visit. After reinforcement, we began to include outpatients from 24 November 2017.

This study was approved by the institutional review board of Seoul National University Hospital (H-1612-041-813). All participants provided written informed consent, and the study was conducted in accordance with the tenets of the Declaration of Helsinki.

### Screening for OSA

The snoring, tiredness, observed apnea, high blood pressure-body mass index, age, neck circumference and gender (STOP-Bang) questionnaire was adopted as a screening tool for OSA in the current study^17^. The STOP-Bang questionnaire has been developed and validated to screen patients for undiagnosed OSA in a preoperative setting. The Korean version of the questionnaire was also validated^18,19^. The questionnaire consists of a total of eight ‘yes’ or ‘no’ questions (snoring, tiredness, observed apnea, high blood pressure, body mass index [BMI] > 35 kg/m^2^, age > 50 years, neck circumference > 40 cm, and the male gender). It has demonstrated a high sensitivity in detecting OSA using a cutoff score of ≥ 3: 84% in detecting OSA at any intensity and 93% in detecting moderate to severe OSA^17^. Recently, an alternative model for scoring the STOP-Bang questionnaire was proposed to improve its specificity in detecting moderate to severe OSA^5^. It classifies participants into three groups based on the STOP-Bang score: low (0–2), intermediate (3–4), and high risk (5–8). Those with STOP-Bang scores of 3 or 4 can be further classified as having a high risk for moderate to severe OSA if they have both a STOP (snoring, tiredness, observed apnea, high blood pressure) score of ≥ 2 and one of the following conditions: (1) BMI > 35 kg/m^2^; (2) neck circumference > 40 cm; or (3) are of the male gender^5^. Questionnaire was handed out and collected by the pulmonology fellows in the procedure preparation room of the dedicated bronchoscopy suite before the procedure. The specific scores of the questionnaire were not disclosed to the attending bronchoscopist and nurse.

### Sedation and monitoring

Our routine practice for flexible bronchoscopy is to use moderate sedation with intravenous midazolam which is administered by an experienced nurse and titrated by the attending bronchoscopist. If required, an adjuvant of 50 mcg of fentanyl is used at the bronchoscopist’s discretion.

All patients were provided with 3 L/min of supplemental oxygen by a nasal cannula at the onset of sedation. The pulse oximetry (Strong-703A,Wuhan Strong Electronics Co., Ltd., China) and electrocardiography were monitored continuously during sedation. As part of standard care, blood pressure was measured before the procedure, every 10 min during the procedure, and right after the procedure. Patient monitoring was performed by an experienced nurse under the direction of the bronchoscopist.

### Cardiopulmonary events and airway maneuvers

The cardiopulmonary events included hypoxemia and hypotension. Hypoxemia was defined as an SpO_2_ of < 90% for any duration, and hypotension was defined as systolic blood pressure of < 90 mm Hg or a decrease of more than 25% from the baseline.

When hypoxemia developed, 10 L/min of supplemental oxygen was routinely administered by a nasal cannula. The need for airway maneuvers such as chin lifts, bag-mask ventilations, or endotracheal intubations was determined by the attending bronchoscopist. The administration of reversal agents for the sedative and the interruption or early termination of a procedure due to cardiopulmonary events were also recorded.

### Statistical analysis

The sample-size calculation was based on a pilot study conducted in our center in November 2016. In our pilot study, 36% of participants with a STOP-Bang score of ≥ 3 experienced cardiopulmonary events, as compared with 19% of those with a STOP-Bang score of < 3.We calculated that an estimated sample of 216 participants would provide the study with at least 80% power to detect a significant difference in the occurrence of cardiopulmonary events with a two-sided significance level of 0.05.

Clinical characteristics were compared between patients with a STOP-Bang score of ≥ 3 and those with a STOP-Bang score of < 3 using the independent samples *t*-test or the Mann–Whitney *U* test for continuous variables. For categorical variables, they were compared using either the χ^2^ test or Fisher’s exact test.

A total dose of midazolam, the use of fentanyl, duration of the procedure, and the total number of procedure types per patient were compared according to the attending bronchoscopists.

To evaluate the association of a STOP-Bang score of ≥ 3 with cardiopulmonary events, multivariable logistic regression was performed with variables selected by the least absolute shrinkage and selection operator (LASSO) method with fivefold cross-validation. LASSO shrinks some coefficients of variables and sets others to 0, and hence tries to retain the good variables of subset selection^20^. In addition, a similar multivariable analysis was conducted adjusting for the same variables selected by the LASSO to examine the association of the alternative scoring model of the STOP-Bang questionnaire with cardiopulmonary events.

*P* values of less than 0.05 were considered significant. Statistical analyses were performed using Stata statistical software (Version 13.1; StataCorp LP, College Station, TX).

### Ethical approval

This study was approved by the institutional review board of Seoul National University Hospital (H-1612-041-813). All participants provided written informed consent, and the study was conducted in accordance with the tenets of the Declaration of Helsinki.

## Results

**Figure 1:**
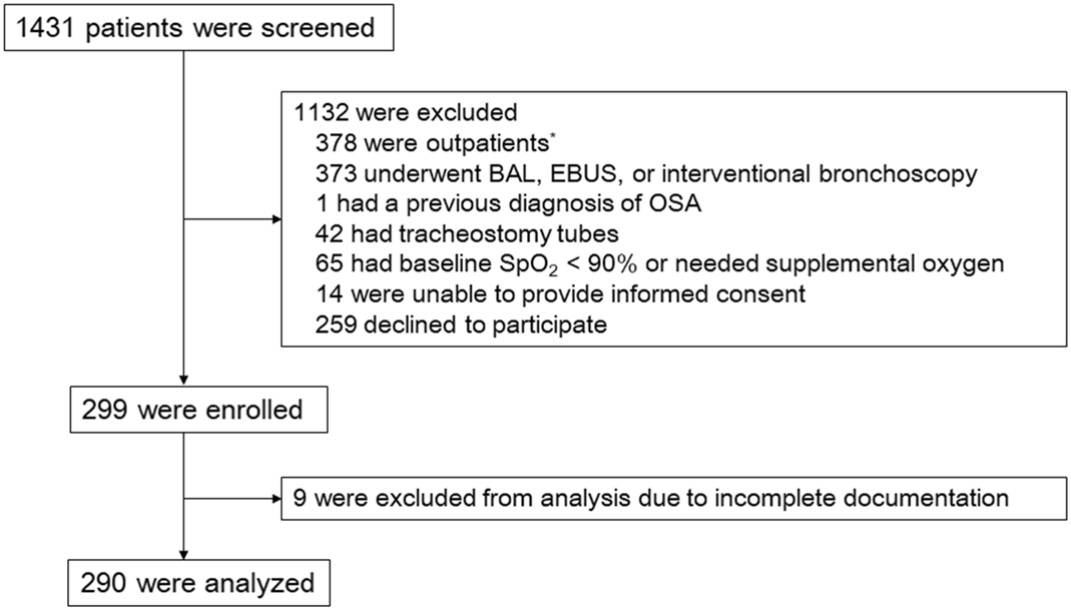
Flowchart of patient selection. *BAL* bronchoalveolar lavage, *EBUS* endobronchial ultrasound, *OSA* obstructive sleep apnea, *SpO*_*2*_ pulse oximeter oxygen saturation. *Initially, outpatients undergoing bronchoscopy were excluded. From 24 November 2017, outpatients were enrolled along with inpatients.

Among 299 patients who were enrolled during the study period, 290 were included in the analysis (Fig. 1). The prevalence of a STOP-Bang score of ≥ 3 was 67.2% (195/290). Table 1 summarizes patient, procedural, and pharmacological characteristics. As expected, patients with a STOP-Bang score of ≥ 3 were significantly older, predominantly male, and had a higher BMI and neck circumference when compared to patients with a STOP-Bang score of < 3. Patients with a STOP-Bang score of ≥ 3 were more likely to be ever-smokers and to have diabetes mellitus and lung cancer as comorbidities. The modified Mallampati score was similar between the two groups. A greater proportion of patients underwent bronchial washing in the group with the STOP-Bang score of < 3; whereas the proportion of patients who underwent an endobronchial biopsy was similar between the groups. The total dose of midazolam and the use of 50 mcg of fentanyl were not significantly different between the groups. Since we started to enroll outpatients undergoing bronchoscopy from the latter period of the study, there were only 9 outpatients. There were no differences between the characteristics of the inpatients and outpatients apart from their median ages (65 [58–73] vs. 58 [55–60]; *P* = 0.01). The prevalence of a STOP-Bang score of ≥ 3 was not significantly different between the inpatients and outpatients (67.3% vs. 66.7%; *P* > 0.99). In addition, a retrospective analysis of 257 patients with spirometry results showed no differences in lung function between patients with a STOP-Bang score of ≥ 3 and those with a score of < 3 (Supplementary Table S1). According to the attending bronchoscopists, there were some differences in the total dose of midazolam, the use of fentanyl, the duration of the procedure, and the total number of procedure types per patient (Supplementary Table S2).

**Table 1:** Characteristics of study patients.

	STOP-Bang < 3 (n = 95)	STOP-Bang ≥ 3 (n = 195)	*P* Value
**Patient characteristics**			
Age, years	65 [56–72]	65 [58–73]	0.14
Male sex	42 (44.2)	140 (71.8)	< 0.001
BMI, kg/m^2^	21.6 ± 2.9	23.7 ± 3.3	< 0.001
Smoking			0.003
Never-smoker	54 (56.8)	72 (36.9)	
Former smoker	22 (23.2)	79 (40.5)	
Current smoker	19 (20.0)	44 (22.6)	
Hospitalization			> 0.99
Inpatients	92 (96.8)	189 (96.9)	
Outpatients	3 (3.2)	6 (3.1)	
ASA physical status			0.09
I	0 (0.0)	0 (0.0)	
II	68 (71.6)	120 (61.5)	
III	27 (28.4)	75 (38.5)	
Comorbidity			
Hypertension	15 (15.8)	113 (58.0)	< 0.001
Diabetes mellitus	8 (8.4)	51 (26.2)	< 0.001
Coronary heart disease	6 (6.3)	19 (9.7)	0.33
Cerebrovascular disease	2 (2.1)	11 (5.6)	0.23
COPD	19 (20.0)	42 (21.5)	0.76
Asthma	4 (4.2)	8 (4.1)	> 0.99
Chronic kidney disease	3 (3.2)	10 (5.1)	0.56
Chronic liver disease	6 (6.3)	7 (3.6)	0.37
Lung cancer	37 (39.0)	105 (53.9)	0.02
Modified Mallampati score			0.78
I	32 (34.0)	58 (30.2)	
II	36 (38.3)	75 (39.1)	
III/IV	26 (27.7)	59 (30.7)	
Neck circumference, cm	33.5 [32.0–36.0]	37.0 [34.5–39.5]	< 0.001
Baseline SBP, mm Hg	127 [117–149]	134 [119–147]	0.28
Baseline heart rate, beats/min	76 [65–82]	72 [65–81]	0.58
Baseline SpO_2_, %	99 [98–100]	99 [97–100]	0.15
Snoring under sedation	22 (23.7)	65 (34.2)	0.07
**Procedural characteristics**			
Sedation time, min	9 [7–12]	10 [7–13]	0.95
Procedure time, min	6 [4–9]	6 [4–9]	0.88
Bronchial washing	78 (82.1)	135 (69.2)	0.02
Endobronchial biopsy	16 (16.8)	38 (19.5)	0.59
Bronchial brushing	0 (0.0)	1 (0.5)	> 0.99
Transbronchial lung biopsy	0 (0.0)	3 (1.5)	0.55
**Pharmacological characteristics**			
Total dose of midazolam, mg	5 [3–5]	5 [3–5]	0.80
Administration of 50 mcg of fentanyl	6 (6.3)	15 (7.7)	0.67

Data are presented as No. (%), mean ± SD, or median [interquartile range].

*ASA* American Society of Anesthesiologists, *BMI* body mass index, *COPD* chronic obstructive pulmonary disease, *SBP* systolic blood pressure, *SpO*_*2*_ pulse oximeter oxygen saturation.

Eighty-one (41.5%) of the 195 patients with a STOP-Bang score of ≥ 3 experienced cardiopulmonary events whereas 26 (27.4%) of the 95 with a STOP-Bang score of < 3 experienced these events (*P* = 0.02; Table 2). The patients with a STOP-Bang score of ≥ 3 tended to experience hypoxemia more than those with a STOP-Bang score of < 3, although the difference was not statistically significant (*P* = 0.052). The lowest SpO_2_ values were the same in both groups (83% [77%–86%] vs. 85% [76%–88%]; *P* = 0.70). There were no cases of bag-mask ventilations or endotracheal intubations. We observed similar rates of cardiopulmonary events requiring reversal agents and interruptions/early terminations of the procedure between the two groups.

**Table 2:** Cardiopulmonary events and airway maneuvers.

	STOP-Bang < 3 (n = 95)	STOP-Bang ≥ 3 (n = 195)	*P* Value
Cardiopulmonary events	26 (27.4)	81 (41.5)	0.02
Cardiopulmonary events (non-exclusive)			
Hypoxemia	26 (27.4)	76 (39.0)	0.052
Hypotension	1 (1.1)	5 (2.6)	0.67
Airway maneuvers			
Chin lift	7 (7.4)	12 (6.2)	0.70
Bag-mask ventilation	0 (0.0)	0 (0.0)	–
Unplanned endotracheal intubation	0 (0.0)	0 (0.0)	–
Administration of reversal agents due to cardiopulmonary events	2 (2.1)	4 (2.1)	> 0.99
Interruption or early termination of a procedure	3 (3.2)	9 (4.6)	0.76

In the univariable logistic regression analysis, the STOP-Bang questionnaire, comorbid hypertension, chronic obstructive pulmonary disease (COPD), and chronic kidney disease, baseline heart rate and SpO_2_, duration of sedation and procedure, and endobronchial biopsy were associated with cardiopulmonary events (Table 3). Lung function of the patients and the attending bronchoscopists were not associated with cardiopulmonary events (Supplementary Table S3 and Table 3). Patients with previous cerebrovascular disease and comorbid chronic liver disease showed tendency of having less cardiopulmonary events (odds ratio [OR], 0.13; 95% confidence interval [CI], 0.02–1.05; Table 3). However, there were only 13 patients with previous cerebrovascular disease and 13 patients with chronic liver disease. Only one patient with previous cerebrovascular disease and one patient with chronic liver disease, whose STOP-Bang score of ≥ 3, experienced cardiopulmonary events.

**Table 3:** Univariable analysis of factors associated with the risk of cardiopulmonary events.

	OR (95% CI)
**STOP-Bang**	
STOP-Bang < 3	1
STOP-Bang ≥ 3	1.89 (1.11–3.21)
STOP-Bang, alternative scoring model	
Low risk	1
Intermediate risk	1.81 (0.99–3.32)
High risk	2.19 (1.19–4.03)
**Patient characteristics**	
Age,/decade	1.22 (0.96–1.54)
Male sex	0.82 (0.50–1.34)
BMI, kg/m^2^	1.02 (0.95–1.09)
Smoking	
Never-smoker	1
Former smoker	1.02 (0.60–1.75)
Current smoker	0.76 (0.40–1.43)
Hospitalization	
Inpatients	1
Outpatients	0.85 (0.21–3.47)
ASA physical status	
II	1
III	1.51 (0.92–2.47)
Comorbidity	
Hypertension	1.80 (1.11–2.91)
Diabetes mellitus	0.85 (0.47–1.55)
Coronary heart disease	0.64 (0.26–1.59)
Cerebrovascular disease	0.13 (0.02–1.05)
COPD	2.47 (1.39–4.39)
Asthma	2.49 (0.77–8.06)
Chronic kidney disease	4.11 (1.23–13.69)
Chronic liver disease	0.13 (0.02–1.05)
Lung cancer	1.40 (0.86–2.25)
Modified Mallampati score	
I	1
II	1.37 (0.78–2.41)
III/IV	0.68 (0.36–1.29)
Neck circumference, cm	1.00 (0.94–1.08)
Baseline SBP, mm Hg	1.00 (0.99–1.02)
Baseline heart rate,/10 beats/min	1.29 (1.08–1.54)
Baseline SpO_2_, %	0.67 (0.57–0.78)
Snoring under sedation	1.24 (0.73–2.08)
**Procedural characteristics**	
Sedation time,/2 min	1.12 (1.02–1.22)
Procedure time,/2 min	1.18 (1.06–1.30)
Bronchial washing	0.82 (0.48–1.41)
Endobronchial biopsy	2.35 (1.29–4.28)
Attending bronchoscopist	
Pulmonologist A	1
Pulmonologist B	0.86 (0.34–2.21)
Pulmonologist C	0.86 (0.24–3.07)
Pulmonologist D	0.43 (0.05–4.02)
Pulmonologist E	0.86 (0.30–2.50)
Pulmonologist F	1.15 (0.56–2.37)
Pulmonologist G	1.51 (0.74–3.08)
Pulmonologist H	0.67 (0.22–2.01)
Pulmonologist I	0.81 (0.30–2.16)
**Pharmacological characteristics**	
Total dose of midazolam, mg	0.89 (0.74–1.06)
Administration of 50 mcg of fentanyl	0.85 (0.33–2.16)

*ASA* American Society of Anesthesiologists, *BMI* body mass index, *COPD* chronic obstructive pulmonary disease, *CI* confidence interval, *OR* odds ratio, *SBP* systolic blood pressure, *SpO*_*2*_ pulse oximeter oxygen saturation.

The logistic LASSO regression selected seven variables, the STOP-Bang questionnaire, comorbid COPD and chronic kidney disease, baseline heart rate and SpO_2_, procedure time, and the history of cerebrovascular disease (Table 4). Although the history of cerebrovascular disease was selected by the LASSO, there was a potential for selection bias because of the exclusion criteria of our study; only ‘healthy’ patients with previous cerebrovascular disease could participate in the study. Therefore, we did a subgroup analysis according to the history of cerebrovascular disease using the same LASSO-selected model. In the subgroup of patients without the history of cerebrovascular disease, a STOP-Bang score of ≥ 3 was significantly associated with cardiopulmonary events (adjusted OR, 1.94; 95% CI, 1.06–3.54; Table 4). Comorbid COPD and chronic kidney disease, low baseline SpO_2_, and long procedure time were also associated with the increased risk of cardiopulmonary events. By using the alternative scoring model of the STOP-Bang questionnaire, being classified at a high risk of having moderate to severe OSA was independently associated with a higher incidence of cardiopulmonary events in the subgroup of patients without the history of cerebrovascular disease (adjusted OR, 2.05; 95% CI, 1.02–4.09; Table 5). The number of patients with the history of cerebrovascular disease was too small to be explored.

**Table 4:** Multivariable analysis of factors associated with the risk of cardiopulmonary events.

	All patients (n = 290)	Patients without a history of cerebrovascular disease (n = 277)
	aOR (95% CI)	aOR (95% CI)
**STOP-Bang**		
STOP-Bang < 3	1	1
STOP-Bang ≥ 3	1.95 (1.07–3.54)	1.94 (1.06–3.54)
**Cerebrovascular disease**		
No	1	–
Yes	0.04 (0.004–0.35)	–
**COPD**		
No	1	1
Yes	2.30 (1.19–4.47)	2.42 (1.24–4.75)
**Chronic kidney disease**		
No	1	1
Yes	5.61 (1.44–21.82)	6.21 (1.49–25.90)
Baseline heart rate,/10 beats/min	1.23 (0.997–1.52)	1.19 (0.96–1.47)
Baseline SpO_2_,/%	0.66 (0.55–0.78)	0.65 (0.54–0.77)
Procedure time,/2 min	1.14 (1.01–1.30)	1.16 (1.02–1.31)

*aOR* adjusted odds ratio, *CI* confidence interval, *COPD* chronic obstructive pulmonary disease, *SpO*_*2*_ pulse oximeter oxygen saturation.

**Table 5:** Multivariable analysis of factors associated with the risk of cardiopulmonary events by using the alternative scoring model of STOP-Bang questionnaire in the subgroup without a history of cerebrovascular disease (n = 277).

	aOR (95% CI)
**STOP-Bang, alternative scoring model**^**a**^	
Low risk	1
Intermediate risk	1.98 (0.996–3.94)
High risk	2.05 (1.02–4.09)
**COPD**	
No	1
Yes	2.44 (1.24–4.79)
**Chronic kidney disease**	
No	1
Yes	6.26 (1.49–26.19)
Baseline heart rate,/10 beats/min	1.19 (0.96–1.47)
Baseline SpO_2_,/%	0.65 (0.54–0.78)
Procedure time,/2 min	1.16 (1.02–1.31)

*aOR* adjusted odds ratio, *CI* confidence interval, *COPD* chronic obstructive pulmonary disease, *SpO*_*2*_ pulse oximeter oxygen saturation.

^a^The alternative scoring model of the STOP-Bang questionnaire classifies participants into three groups based on the STOP-Bang score: low (0–2), intermediate (3–4), and high risk (5–8). Those with STOP-Bang scores of 3 or 4 can be further classified as having a high risk for moderate to severe OSA if they have both a STOP (snoring, tiredness, observed apnea, high blood pressure) score of ≥ 2 and one of the following conditions: (1) BMI > 35 kg/m^2^; (2) neck circumference > 40 cm; or (3) are of the male gender.

## Discussion

In the present study, we found that two thirds of the patients undergoing bronchoscopy under moderate sedation were classified as being at a high risk of having OSA based on the STOP-Bang questionnaire. The STOP-Bang questionnaire can predict cardiopulmonary events during the procedure although the incidences of cardiopulmonary events requiring airway maneuvers or the termination of the procedure were low and did not increase in these patients.

Intravenous sedation should be provided to patients who have to undergo flexible bronchoscopy and who do not have contraindications to sedative agents^16^. Intravenous midazolam is the preferred sedative for bronchoscopy given its relatively rapid onset of action and its shorter half-life compared to other benzodiazepines. Midazolam can result in respiratory depression, in part, by increasing the activity of γ–aminobutyric acid, the major inhibitory neurotransmitter in the brain^21^. In addition, midazolam administered in sedative doses can increase supraglottic airway resistance leading to obstructive apnea^22^. Moreover, the addition of opioids to midazolam can increase the risk of respiratory depression although it improves the patient’s procedural tolerance^16,23^.

However, the incidences of adverse events occurring in patients with undiagnosed OSA, undergoing bronchoscopy with intravenous sedation, remain poorly identified. Recently, May et al*.* have reported that there was no association between the STOP-Bang questionnaire and respiratory complications during bronchoscopy under moderate sedation^24^. In their study, age, the need for oxygen supplementation at baseline, and procedure duration were associated with respiratory complications. Several differences in study designs could explain the discrepancies between the two studies. First, to reduce the effects of procedure itself on respiratory complications, we excluded patients undergoing BAL, advanced diagnostic and therapeutic flexible bronchoscopy. BAL is one of the common causes of hypoxemia during bronchoscopy along with upper airway obstruction, interventional bronchoscopic procedures, oversedation or inadequate sedation, inadequate oxygen supplementation, bleeding, and laryngospasm^25^. In the study by May et al*.*, proportions of patients who underwent BAL, EBUS, and interventional bronchoscopy were 62.8%, 45.3%, 3.6%, respectively. As expected, EBUS had the highest OR of respiratory complications in their univariable analysis. By excluding patients with BAL, EBUS, interventional bronchoscopy, the median procedure time (6 min vs. 30 min) was shorter and there was lower mean number of procedure types (1.9 per patient vs. 2.4 per patient) in our study compared to the previous study. Second, we also excluded patients with hypoxemia or the need for supplemental oxygen before the procedure. As the previous study included patients in need of baseline oxygen supplementation, respiratory complication during the procedure may be caused and aggravated by their underlying conditions. By excluding patients with hypoxemia at baseline or undergoing BAL, advanced diagnostic and therapeutic bronchoscopy, we tried to focus on respiratory complications caused by upper airway obstruction under moderate sedation. Third, the outcome measure was different. The outcome of the previous study was a composite of respiratory complications of hypoxemia (SpO_2_ ≤ 85%), bradypnea, the administration of reversal agents for the sedative, airway maneuvers, and premature termination of the procedure. In contrast, the cardiopulmonary events in this study was hypoxemia and hypotension, which are not dependent on attending bronchoscopists and nurses.

In our study, the reverse causation phenomenon between the history of cerebrovascular disease and cardiopulmonary events during a bronchoscopy was observed. This could be explained by the healthy worker effect. As we excluded patients with ASA physical status of > 3, a tracheostomy tube, hypoxemia or the need for supplemental oxygen at baseline, and an inability to provide informed consent, stronger selection of relatively healthy patients with previous cerebrovascular disease occurred. In addition, bronchoscopists and nurses might have been more cautious in performing bronchoscopy in those patients, which could have contributed to reverse causation. Consequently, the subgroup analysis was performed across the subgroups with or without the history of cerebrovascular disease. In the subgroup of patients without the history of cerebrovascular disease, cardiopulmonary events occurred at an increased frequency during bronchoscopy in patients at high risk of having OSA.

In relation to the Bradford Hill criteria^26^, we found a weak dose–response relationship between the degree of the risk of having OSA and cardiopulmonary events when the alternative scoring model of the STOP-Bang questionnaire was applied: the increased risk of OSA was associated with the increased risk of cardiopulmonary events during bronchoscopy under moderate sedation. This supports the biological plausibility of our findings.

Patients with COPD and chronic kidney disease showed an increased risk of experiencing cardiopulmonary events during a bronchoscopy. While bronchoscopy is generally safe in most patients with COPD^27^, patients with severe to very severe COPD could have more frequent respiratory complications than patients without COPD^28^. In addition, OSA coexisting with COPD, called COPD-OSA overlap syndrome^29^, might affect the risk of cardiopulmonary events occurring during bronchoscopy. Although studies regarding the safety of bronchoscopy in patients with chronic kidney disease are scarce^30,31^, the elimination of midazolam and its metabolite is reduced in patients with a renal impairment^32^. Moreover, the fluid overload and rostral fluid shift occurring in the supine position might contribute to upper airway narrowing and thus, increase the risk of OSA in those patients^33^.

To appreciate the results of our study appropriately, we have to recognize its limitations. First, capnography monitoring was not applied, and thus, bradypnea, hypopnea, and hypercapnia were not counted as a cardiopulmonary event. Detection of hypercapnia by capnography may precede hypoxemia, especially in cases where supplemental oxygen may mask hypoventilation by delaying oxygen desaturation. Second, we were not able to confirm the diagnosis of OSA using polysomnography in patients with a STOP-Bang score of ≥ 3. However, the STOP-Bang questionnaire has been widely adopted and validated in various populations showing a high sensitivity in detecting moderate to severe OSA^5^. Third, although the specific scores of the STOP-Bang questionnaire were not disclosed to the attending bronchoscopist and nurse, the age, sex, and BMI, which are part of the questionnaire, could influence the administration of sedatives and the levels of intervention needed.

In conclusion, two thirds of the patients undergoing bronchoscopy under moderate sedation were at risk of having OSA based on the STOP-Bang questionnaire. Using the simple and validated screening questionnaire can help identify patients with the increased risk of cardiopulmonary events occurring during bronchoscopy under moderate sedation.

## Supplementary information

Supplementary Information.

## Data Availability

The datasets generated during and/or analyzed during the current study are available from the corresponding author on reasonable request.
