# Supplementary material for: Prediction of cardiopulmonary events using the STOP-Bang questionnaire in patients undergoing bronchoscopy with moderate sedation
Source: Sci Rep. 2020 Sep 2;10:14471. doi: 10.1038/s41598-020-71314-1 (PMC7468304; doi:10.1038/s41598-020-71314-1)
Supplement: Supplementary file 1 — Supplementary Information. [file 41598_2020_71314_MOESM1_ESM.docx]

**Prediction of cardiopulmonary events using the STOP-Bang questionnaire in patients undergoing bronchoscopy with moderate sedation**

Jaeyoung Cho, MD, Sun Mi Choi, MD, Young Sik Park, MD, Chang-Hoon Lee, MD, PhD, Sang-Min Lee, MD, PhD, Chul-Gyu Yoo, MD, PhD, Young Whan Kim, MD, PhD, Jinwoo Lee, MD

**S1 Table.** Spirometry results of 257 study patients

|  | STOP-Bang < 3  (n = 84) | STOP-Bang ≥ 3  (n = 173) | *P* Value |
| --- | --- | --- | --- |
| FEV_1_/FVC, % | 74 [64–79] | 71 [65–79] | 0.92 |
| FEV_1,_ % predicted | 92 ± 24 | 96 ± 22 | 0.12 |
| FVC, % predicted | 92 [80–108] | 98 [84–107] | 0.38 |

FEV_1_, forced expiratory volume in 1 second; FVC, forced vital capacity.

**S2 Table.** Procedural and pharmacological characteristics of study patients according to attending bronchoscopists

|  | Pulmonologist | | | | | | | | | *P* value |
| --- | --- | --- | --- | --- | --- | --- | --- | --- | --- | --- |
|  | A | B | C | D | E | F | G | H | I |  |
| The number of bronchoscopies | 101 | 24 | 12 | 5 | 18 | 45 | 45 | 18 | 22 |  |
| Total dose of midazolam, mg | 5 [3–5] | 4 [3–5] | 3 [3–4] | 4 [3–4] | 5 [4–5] | 5 [4–5] | 4 [3–5] | 5 [3–5] | 6 [5–7] | < 0.001 |
| Administration of 50 mcg of fentanyl | 0 (0.0) | 20 (83.3) | 0 (0.0) | 0 (0.0) | 0 (0.0) | 0 (0.0) | 0 (0.0) | 0 (0.0) | 1 (4.6) | < 0.001 |
| Sedation time, min | 9 [7–12] | 8 [6–10] | 11 [8–15] | 10 [9–13] | 6 [6–8] | 11[8–14] | 10 [8–12] | 11 [7–14] | 12 [10–19] | < 0.001 |
| Procedure time, min | 5 [4–8] | 5 [3–6] | 7 [5–13] | 6 [5–6] | 4 [2–4] | 8 [6–11] | 6 [4–8] | 6 [3–9] | 8 [5–16] | < 0.001 |
| The total number of procedure types, /patient^*^ | 2.0 ± 0.6 | 1.8 ± 0.6 | 2.2 ± 0.6 | 1.8 ± 0.8 | 1.5 ± 0.7 | 1.8 ± 0.6 | 2.0 ± 0.6 | 2.0 ± 0.7 | 2.1 ± 0.5 | 0.02 |

^*^The total number of procedure types is the sum of any procedures of airway exam, bronchial washing and brushing, endobronchial biopsy, and transbronchial lung biopsy.

**S3 Table.** Univariable analysis of the relationship between lung function and cardiopulmonary events

|  | OR (95% CI) |
| --- | --- |
| FEV_1_/FVC, % | 0.99 (0.97–1.01) |
| FEV_1,_ % predicted | 0.99 (0.98–1.01) |
| FVC, % predicted | 1.00 (0.98–1.01) |

CI, confidence interval; FEV_1_, forced expiratory volume in 1 second; FVC, forced vital capacity; OR, odds ratio.
